# The association between gout and cataract risk: A meta-analysis

**DOI:** 10.1371/journal.pone.0180188

**Published:** 2017-06-29

**Authors:** Chenqi Luo, Xinyi Chen, Hongchuan Jin, Ke Yao

**Affiliations:** 1 Eye Center, Second Affiliated Hospital, School of Medicine, Zhejiang University, Hangzhou, China; 2 Department of Medical Oncology, Key Laboratory of Biotherapy in Zhejiang, Sir Runrun Shaw Hospital, Medical School of Zhejiang University, Hangzhou, China; Tsinghua University School of Life Sciences, CHINA

## Abstract

**Purpose:**

To evaluate the relationship between gout and age-related cataracts (ARCs).

**Methods:**

A comprehensive literature search of the PubMed and Web of Science databases was conducted to identify papers on the association between gout and cataract risk that had been published between February 1991 and January 2017. Pooled relative risks (RRs) or odds ratios (ORs) and their corresponding 95% confidence intervals (CIs) were calculated. The random-effects model was used instead of the fixed-effects model when heterogeneity was identified, as indicated by a Cochran’s Q statistic P-value <0.10 or I2 index score >50%.

**Results:**

A total of 3 cross-sectional studies and 3 case-control studies were included in the meta-analysis. Gout was significantly associated with increased odds of ARCs (OR 1.53, 95% CI 1.27–1.84). In the subgroup analysis, gout exhibited positive associations with the odds of posterior subcapsular cataracts (PSCs, OR 1.69, 95% CI: 1.06–2.70) and cortical cataracts (CCs, OR 1.39, 95% CI: 1.06–1.81). However, no association was identified between gout and the odds of nuclear cataracts.

**Conclusions:**

The current literature suggested that gout may be associated with increased odds of ARCs, especially PSCs and CCs. Further efforts should be made to confirm these findings and clarify the effect of gout and gout medications on the development of cataracts.

## Introduction

Cataracts are a major cause of visual impairment and blindness in older adults worldwide [1]. With the rapidly aging population, cataracts are becoming a significant social problem in the global context. In addition to advancing age, other factors that have been reported to increase the risk of cataracts include sunlight exposure, alcohol consumption, smoking and some medications [2, 3].

In addition, some metabolic diseases have been reported to be associated with cataract formation (e.g., diabetes mellitus, galactosemia and tetany). Gout is a chronic disease characterized by the deposition of monosodium urate crystals, which form in the presence of increased urate concentrations [4]. Previous studies have focused on the association between gout and cataract formation. However, the results of these studies have been inconclusive. Some studies have demonstrated that gout may be associated with increased risk of developing cataracts [5, 6]. In contrast, several studies have reported the presence of no association between gout and cataract development [7]. These individual studies may be restricted in terms of sample size. Therefore, in the present investigation, a meta-analysis of published observational studies was performed to analyze the relationship between gout and cataract prevalence.

## Materials and methods

### Search strategy and selection of papers

A meta-analysis was performed according to standard methods for systematic reviews and meta-analyses [8–10]. The PubMed and Web of Science databases were searched to identify original papers on the relationship between gout and cataract risk that had been published between February 1991 and January 2017. The search strategy comprised terms related to cataracts (“cataract”, “lens opacity”, “crystalline opacity”) and gout (“gout”, “gout therapy”), and only human studies were included. Furthermore, the references of selected papers were manually searched for potentially relevant papers. First, two independent reviewers conducted a preliminary review of the titles and abstracts of identified articles; then, two investigators independently screened the full texts of all selected studies using the inclusion criteria described below. The studies were required to meet the following criteria: (1) original research papers directly reporting study results; and (2) cross-sectional or case-control studies estimating the influence of gout on cataract risk using odds ratios (ORs) or relative risks (RRs) and their corresponding 95% confidence intervals (CIs). We excluded papers based on the following criteria: (1) non-original papers (“reviews”, “letters”, “comments”, etc.); (2) non-age-adjusted studies, as age is considered the most reliable independent risk factor for cataracts; and (3) duplicate publications, as only the most recent or most informative studies were included.

### Data extraction and quality assessment

The following data were independently extracted from the included studies by two authors (Chenqi Luo and Xinyi Chen): first author name, publication year, country, study design, sample size, age, cataract definitions and subtypes (nuclear, cortical, or posterior subcapsular), gout status, control variables, and OR/RR values with 95% CIs. Then, the two authors compared the extraction results to identify differences; conflicting evaluations were discussed among all study authors, and discrepancies were resolved by achieving consensus. If more than one adjusted model was included in a single study, only the model in which the OR/RR values were most fully adjusted for potential confounding variables was selected [11]. Gout and cataracts were diagnosed by physicians or study researchers. Diagnostic criteria used for cataracts included the Lens Opacities Classification System (LOCS) I—III, Age-Related Eye Disease Study (AREDS) criteria, Wisconsin Cataract Grading System and Wilmer Cataract Grading System. In addition, the associations between gout and the subtypes of cataracts were assessed. The Newcastle-Ottawa Scale (NOS) was used for quality assessment. A maximum of nine stars can be assigned on this scale, which is organized as follows: four stars possible in the patient selection category, two stars possible in the comparability category, and three stars possible in the outcome assessment category. Scores ≥7 indicate good quality.

### Statistical methods

OR/RR values and their corresponding 95% CIs were extracted from all qualified studies and summarized to obtain pooled ORs/RRs. Subgroup analyses were conducted based on cataract subtype. The level of heterogeneity across individual studies was evaluated using the Cochran’s Q statistic and I^2^ index score, with the level of significance set at a *P*-value <0.10 or I^2^ score >50%, respectively [12]. The fixed-effects model (the inverse variance method [13]) was used when no heterogeneity was observed across the included studies. Otherwise, the random-effects model (DerSimonian and Laird method [14]) was used. A sensitivity analysis was performed to evaluate the robustness of the meta-analysis estimates, and meta-regression analyses were conducted to explore the potential sources of heterogeneity. Publication bias was assessed using the Egger’s linear regression test [15] and Begg’s rank correlation test [16]. The statistical software used for the analyses was STATA version 11.0 (STATA Corporation, College Station, TX), and the significance level was set to *P* < 0.05.

## Results

### Characteristics of included studies

**Fig 1:**
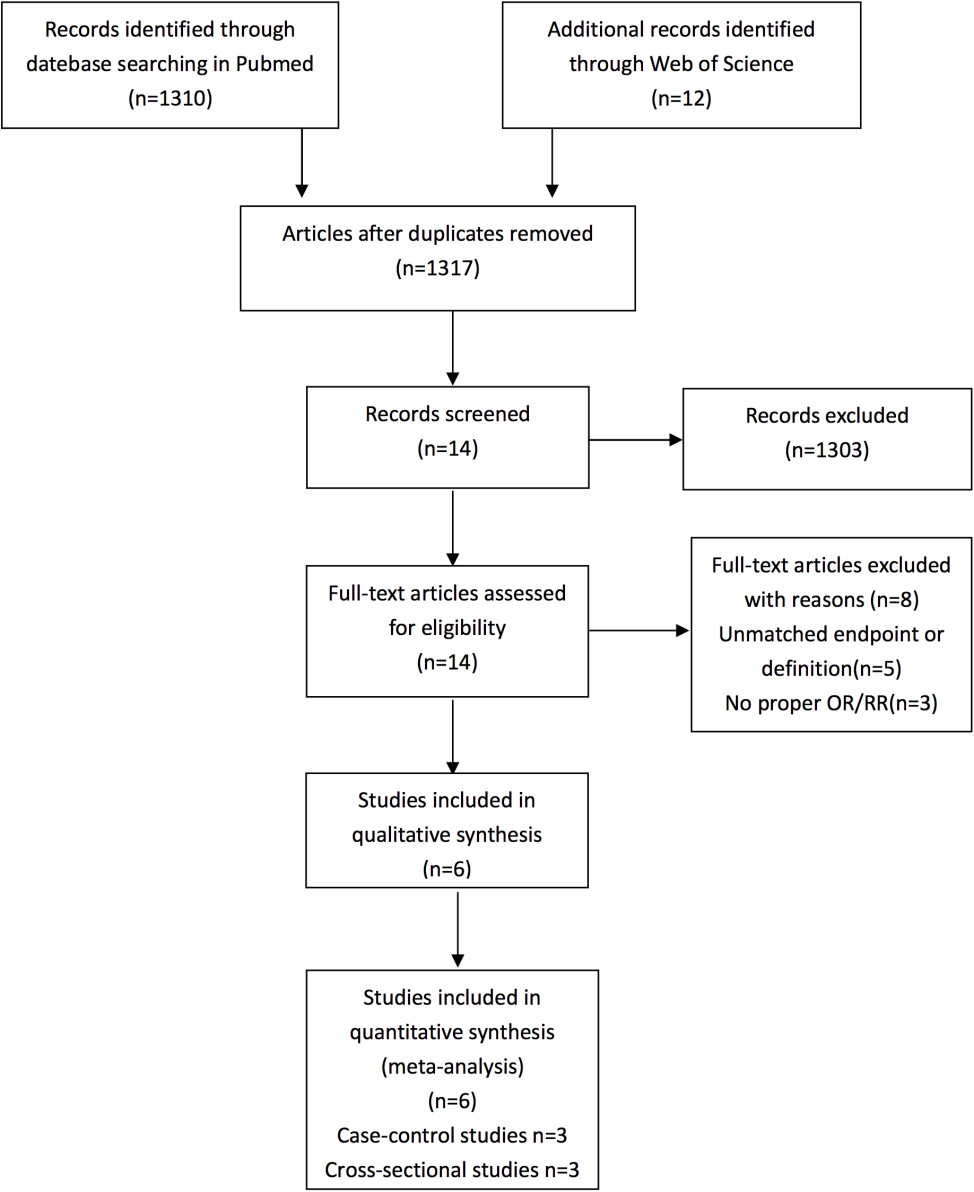
Flow diagram of the literature search in this meta-analysis.

The flowchart in Fig 1 shows the literature search process. After duplicates were removed, the titles and abstracts of 1317 potentially relevant articles were scanned, and 1303 studies were excluded. Fourteen full-text articles were then assessed for eligibility. Eight articles were excluded for following reasons: 3 articles did not provide appropriate ORs/RRs and 5 studies used inconsistent endpoints or definitions. Finally, 6 [5, 7, 17–20] studies meeting all of the predefined inclusion criteria were included in the present meta-analysis.

The characteristics of the included studies are presented in Table 1. Of the 6 included studies, three were cross-sectional in nature and the other three utilized a case-control design. The results of the quality assessment of the studies are displayed in S1 PRISMA Checklist. According to the results identified using the NOS scale, 6 of the included studies were considered high quality.

**Table 1:** Characteristics of 6 studies included into present meta-analysis.

Source (Published Year, Country)	Study Design	Race	Sample Size	Age (year)	Cataract types	Cataract Definition	Gout Diagnosis	Adjusted Variables	NOS
Catherine Anne McCart (2000Australia)	Population- based cross-sectional	Caucasian	4744	≥40	CC	Standard criteria	Confirmed by study researcher	Age,gender, diabetic status,arthritis status, refractive status,UV-B exposure, family history	7
Bickol N. Mukesh (2006Australia)	Population- based cross-sectional	Caucasian	3721	40–98	NC	Standard criteria	Confirmed by study researcher	age	8
Bickol N. Mukesh (2006Australia)	Population- based cross-sectional	Caucasian	3721	40–98	PSC	Standard criteria	Confirmed by study researcher	age	8
George S. Zubenko (2007 America)	Population- based case-control	Caucasian	100	≥90	Any type, NC,CC,PSC	Standard criteria	Confirmed by study researcher	Age,sex, Education, Diabetes Dmellitus, Hypertension,Arthritis, Steroid use,Smoking,drinking	7
M. Cristina Leske (1998 America)	Population- based case-control	Caucasian	764	≥40	NC	Lens opacity impairing vision	Confirmed by study researcher	Gender,age, education,race,smoking,Family history	8
Cathy A. Mccarty(1999Australia)	Population- based cross-sectional	Caucasian	5147	≥40	CC	Standard criteria	Confirmed by study researcher	Age, female gender, diabetes, arthritis, myopia,use of oral beta-blockers, ultraviolet B exposure	8
Cathy A. Mccarty (1999Australia)	Population- based cross-sectional	Caucasian	5147	≥40	CC	Standard criteria	Confirmed by study researcher	Age,female gender, diabetes,arthritis,myopia,use of oral beta-blockers, ultraviolet B exposure	8
M.CristinaLeske (1991America)	hospital-base case-control study	Caucasian	1380	40–79	PSC	Standard criteria	Confirmed by Ophthalmologist	Age,sex	8
M.CristinaLeske (1991America)	hospital-base case-control study	Caucasian	1380	40–79	CC	Standard criteria	Confirmed by Ophthalmologist	Age,sex	8
M.CristinaLeske (1991America)	hospital-base case-control study	Caucasian	1380	40–79	NC	Standard criteria	Confirmed by Ophthalmologist	Age,sex	8
M.CristinaLeske (1991America)	hospital-base case-control study	Caucasian	1380	40–79	Any type, NC,CC,PSC	Standard criteria	Confirmed by Ophthalmologist	Age,sex	8

CC: cortical cataract; PSC: posterior subcapsular cataract; NC:nuclear cataract.

### Association between gout and cataract risk

**Fig 2:**
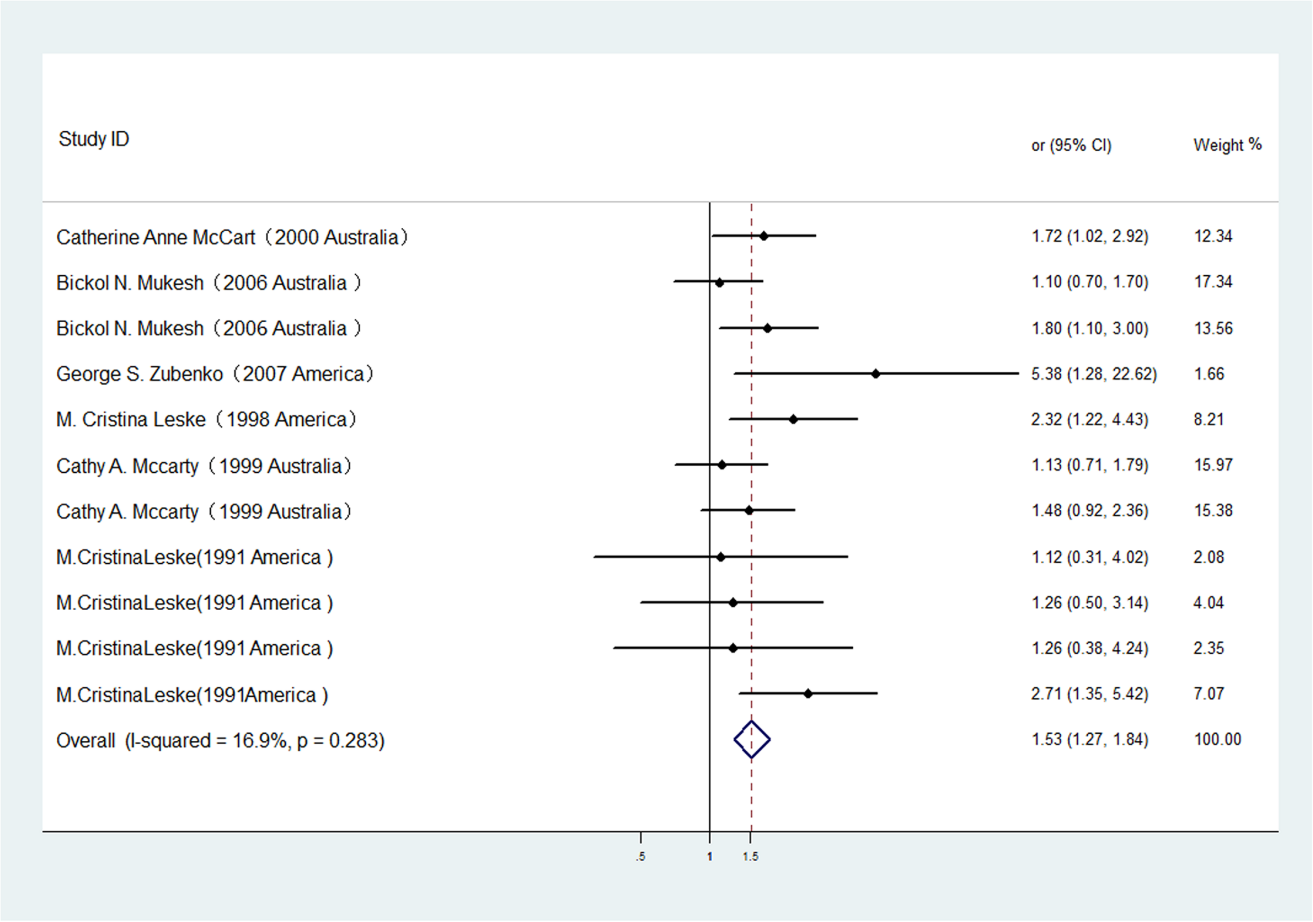
The association of gout with cataract risk in case-control/cross-sectional studies.

The studies included in the meta-analysis assessed the association between gout and the risk of any type of cataract. Fig 2 shows the significantly greater odds of any type of cataract that were identified in patients with gout vs. patients without gout using the random-effects model (OR 1.53, 95% CI 1.27–1.84). No significant heterogeneity was observed (p = 0.283, I2 = 16.9%). A sensitivity analysis was conducted by excluding one study per iteration. The exclusion of any single study did not alter the overall pooled result, indicating robust meta-analysis results. In addition, no significant publication bias was observed in the selected 6 studies (Begg's test, p = 0.386; Egger's test, p = 0.215).

**Fig 3:**
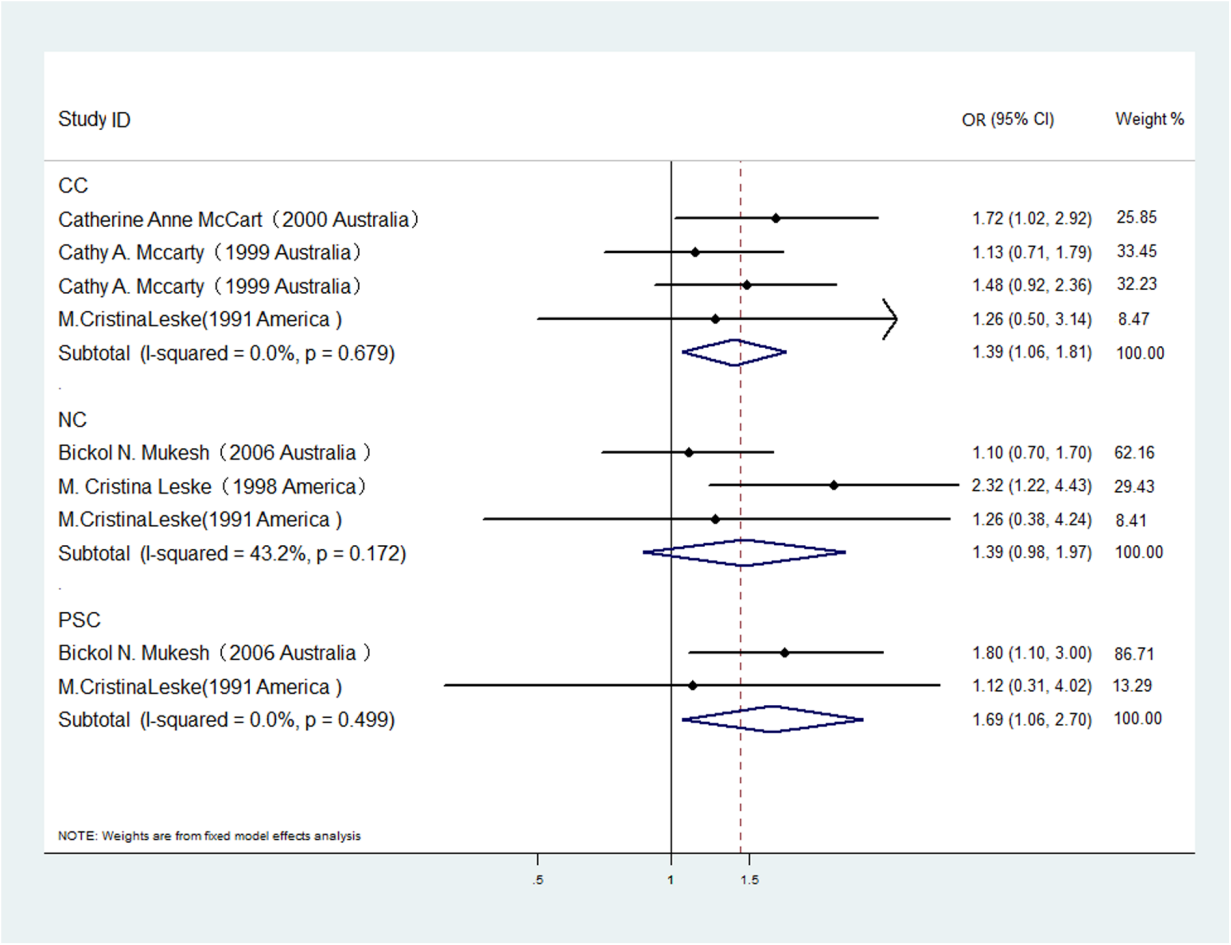
The association of gout with risk of cataract subtypes in case-control/cross-sectional studies.

A subgroup analysis was also conducted to investigate the effect of cataract subtype on the relationship between gout and cataract odds (Fig 3). Three[7, 17, 18], three[5, 18, 19] and two[5, 18] studies were included in the analysis of the associations between gout with cortical, nuclear and posterior subcapsular cataract odds, respectively. The pooled estimates indicated that hypertension significantly increased the odds of posterior subcapsular cataracts (OR 1.69, 95% CI: 1.06–2.70; I2 = 0%, *P heterogeneity* = 0.499) and cortical cataracts (OR 1.39, 95% CI: 1.06–1.81; I2 = 0%, *P heterogeneity* = 0.679) but did not increase the odds of nuclear cataracts (OR 1.39, 95% CI: 0.98–1.97; I2 = 43.2%, *P heterogeneity* = 0.172). No publication bias was observed among the studies included in each subgroup described above.

## Discussion

Our meta-analysis showed that gout was associated with increased odds of ARCs in cross-sectional studies and case-control studies. A positive association was also identified in the analyses stratified by type of cataract (cortical cataracts (CCs) or posterior subcapsular cataracts (PSCs)). However, no association was observed between gout and nuclear cataracts in the included cross-sectional and case-control studies.

As expected based on the results of previous population studies [21, 22], gout and its treatments were considered as risk factors for ARC development. Allopurinol and colchicine are antihyperuricemic agents commonly used to treat gout.

Colchicine has been used to treat gout for centuries and is the most specific agent available for acute gout because its effects are targeted toward factors prominent in crystal‐induced inflammation. Colchicine has been reported to cause lethal cell injury via microtubule disruption in a process that is readily distinguished from two well-known pathways that lead to a loss of viability, namely, oxidative stress and inhibition of mitochondrial electron transport. Besides, colchicine-induced cell death has been found to be accompanied by DNA fragmentation[23]. In addition, colchicine may also influence the odds of lens opacification through these mechanisms.[20]

Previous studies have reported that long-term administration of allopurinol increases the odds of cataract extraction in elderly patients.[24] Another study showed long-term ingestion of allopurinol to be related to the development of lens opacity in relatively young patients. [25] Lerman et.al[26] reported that allopurinol only had cataractogenic activity n patients in whom the drug had become photobound within the lens, which indicates the influence of the relationship between ultraviolet radiation exposure and circulating allopurinol levels in the genesis of photosensitized allopurinol cataracts. Ultraviolet radiation, even at ambient levels, has been implicated in permanently photobinding allopurinol to lens proteins. Authors have hypothesized that bound allopurinol absorbs nearby ultraviolet radiation, which is then transmitted through the cornea, thereby enhancing the “normal, actinic, aging” process in the lens.[27] The cataractogenic potential of allopurinol, especially for cortical cataracts (CCs) and posterior subcapsular cataracts (PSCs) development, has been suggested in some reports.[28] The cataracts associated with this antihyperuricemic agent have been found to initially occur in the form of anterior and posterior lens capsule changes with anterior subcapsular vacuoles. Over time, wedge-shaped anterior and posterior cortical haze occurs and dense posterior subcapsular cataracts develop.[28] Liu et al [27] reported that unusual morphological thinning of the anterior clear zone of the lens may be observed in gout patients on long-term allopurinol therapy. In addition, clinical data suggest that there may be an association between the development of anterior subcapsular cataracts and thinning of the anterior clear zone.

The aforementioned studies were conducted to evaluate the association between gout medications and cataracts; however, gout itself may have an effect on cataract development. McCarty et al [7] found that a gout duration of more than 10 years was associated with increased odds of cortical cataracts in all age groups except the youngest.

Inevitably, the present meta-analysis has several limitations that may affect the interpretation of its results. The first limitation stems from the absence of clinical trials. Because gout status is determined naturally, it was impossible to randomly divide the participants into gout patients and non-gout patients. Therefore, RCTs cannot be applied for this type of analysis. As a consequence, cross-sectional studies and case-control studies, which are not as reliable as RCTs, were included in this meta-analysis. Second, the assessment of cataracts and control factors varied across the included studies, resulting in increased heterogeneity. Finally, we could not verify whether gout itself or the gout medications affected cataracts, more studies about the mechanism were needed.

Our meta-analysis had several strengths. Each study was adjusted for age, which has been identified as the most reliable independent risk factor for cataracts. Most studies included in our meta-analysis were based on the general population, resulting in increased generalizability. In addition, we performed a subgroup analysis based on cataract type to separately analyze the relationships between different cataract types and gout.

## Conclusions

In our meta-analysis of cross-sectional and case-control studies, we summarized risk estimates for the association between gout and ARCs, especially CCs and PSCs, and provided robust evidence of this association. These data may help to resolve some of the inconsistencies in the relationship between gout and the odds of ARCs, and future research is needed to confirm these findings.

## Supporting information

S1 PRISMA Checklist PRISMA 2009 checklist in this meta-analysis. (DOC)
